# Assessing Genetic Structure and Diversity Using Multiple Molecular Markers to Guide Conservation Management of *Saussurea involucrata* in the Eastern Tianshan Mountains

**DOI:** 10.3390/ijms27052274

**Published:** 2026-02-28

**Authors:** Jiayi Lu, Kelimunur Maimaiti, Jingdian Liu, Daoyuan Zhang, Dunyan Tan, Jiancheng Wang, Wei Shi

**Affiliations:** 1 College of Forestry and Landscape Architecture, Xinjiang Agricultural University, Urumqi 830011, China; lujiayi00971@163.com (J.L.); kel13619906253@126.com (K.M.); ariiiiiink@gmail.com (J.L.); tandunyan@163.com (D.T.); 2 State Key Laboratory of Desert and Oasis Ecology, Key Laboratory of Ecological Safety and Sustainable, Development in Arid Lands, Xinjiang Institute of Ecology and Geography, Chinese Academy of Sciences, Xinjiang 830011, China; zhangdy@ms.xjb.ac.cn; 3 Turpan Eremophytes Botanical Garden, Chinese Academy of Sciences, Turpan 838008, China; 4 Xinjiang Key Lab of Conservation and Utilization of Plant Gene Resources, Xinjiang Institute of Ecology and Geography, Chinese Academy of Sciences, Urumqi 830011, China

**Keywords:** *Saussurea involucrata*, conservation genetics, management unit

## Abstract

*Saussurea involucrata* is a rare perennial herb endemic to the alpine zone of the Tianshan Mountains, possessing significant medicinal value yet facing severe threats from overharvesting and habitat fragmentation. The core distribution area and recognized genetic differentiation center of this species are located in the Bayinbuluke region of the Western Tianshan Mountains. In contrast, the genetic distinctiveness and conservation status of populations in the Eastern Tianshan Mountains have remained unclear. To clarify the genetic relationships, we conducted an integrated analysis using nuclear microsatellite (SSR) markers as well as chloroplast (*cp*DNA) and nuclear ribosomal DNA (nrDNA) sequences on 16 populations (5 from the Eastern Tianshan Mountains and 11 from the Bayinbuluke region). The results showed that the Eastern Tianshan Mountains populations exhibited higher genetic diversity (mean *He* = 0.5568). Bayesian clustering and principal coordinate analysis (PCoA) clearly separated all populations into two genetic groups corresponding to the two geographical regions. Notably, private haplotypes (*cp*DNA H1 and nrDNA H7) were identified exclusively in the Eastern Tianshan populations, and no recent genetic bottleneck was detected, indicating historical demographic stability. These findings demonstrate significant genetic differentiation and a unique evolutionary trajectory in the Eastern Tianshan Mountains populations, likely resulting from long-term geographical isolation and local adaptation to arid environments. Therefore, we propose that these populations be managed as an independent Management Unit (MU) to preserve their unique genetic legacy. This study provides critical genetic evidence for refining conservation strategies and promoting the sustainable use of this endangered species, while the established molecular marker system also offers a reliable framework for its geographical traceability.

## 1. Introduction

The conservation of endangered species represents a critical challenge that necessitates a multidisciplinary approach. In this endeavor, the fields of conservation genetics and population ecology have become indispensable, providing the scientific foundation for diagnosing threats and formulating effective recovery strategies [1]. Conservation genetics specifically aims to reveal the genetic mechanisms underlying population decline and extinction risk in endangered species. By elucidating patterns of genetic diversity, structure, and gene flow, this discipline offers a theoretical framework for developing scientifically sound and actionable protection plans [2]. Assessing the genetic diversity of an endangered species constitutes the fundamental first step in evaluating its current conservation status [3,4,5]. Such an assessment not only informs our understanding of its present viability but also aids in predicting potential future evolutionary trajectories and in designing interventions to prevent further population decline. Ultimately, genetic diversity serves as the essential substrate for species survival, adaptation, and long-term evolutionary resilience.

*Saussurea involucrata* (Kar. & Kir.) Sch. Bip., a perennial herb within the Asteraceae family, exemplifies a species facing severe conservation threats. Its distribution is primarily confined to the alpine zones of the Tianshan Mountains, with a major concentration in the western ranges. Valued for centuries in traditional medicine, *S. involucrata* demonstrates notable pharmacological effects against inflammation, oxidative stress, fatigue, and rheumatic diseases [6]. This very utility, however, has led to its severe overexploitation through excessive collection from the wild. Compounding this anthropogenic pressure are the species’ intrinsic biological constraints. *S. involucrata* requires extremely stringent environmental conditions, with its distribution tightly restricted to narrow habitats adjacent to the alpine snowline. Furthermore, global climate change is causing rapid glacial retreat and snowline elevation in its mountain habitat [7], leading to continual diminishment and fragmentation of its already limited niche. In recent decades, the synergistic effects of overharvesting and climate-driven habitat degradation have resulted in a significant decline in natural populations, drastically elevating its risk of extinction [8]. The species is further hampered by slow growth and limited natural regeneration capacity, making population recovery from disturbance exceptionally difficult. In recognition of its precarious state, *S. involucrata* is listed as a nationally protected plant of Class II in China.

Research on *S. involucrata* spans several areas, including phytochemistry, pharmacology [9,10], and the development of artificial cultivation techniques [11,12]. Significant attention has also been directed toward its conservation genetics. For instance, an early study by Shi analyzed five populations from the Western Tianshan Mountains, identifying 12 *cp*DNA and 5 nrDNA haplotypes [13]. This work revealed high genetic diversity and found no evidence of recent population bottlenecks or rapid expansion. Expanding the geographical scope, Hu examined nine populations across the Tianshan and Altay Mountains, discovering 12 chloroplast and 7 nuclear haplotypes that delineated two separate lineages [14]. This study proposed the Bayinbuluke area as a potential center of origin and genetic divergence for the species. A subsequent, finer-scale analysis by Hu, utilizing 18 SSR markers on 112 individuals from 11 Bayinbuluke populations, further confirmed high genetic diversity within this key region [15].

Despite these advancements, a significant geographical bias persists in the genetic research on *S. involucrata*. Previous studies have predominantly focused on Central and Western Tianshan populations, paying insufficient attention to those in the Eastern Tianshan Mountains. Consequently, genetic information for these Eastern Tianshan Mountains populations remains scarce, creating a critical knowledge gap that limits the development of comprehensive, range-wide conservation strategies. To address this gap, our study focuses on the genetic diversity and structure of *S. involucrata* populations in the Eastern Tianshan Mountains, using the well-characterized Bayinbuluke region as a reference. Our findings aim to provide concrete suggestions for the targeted protection of these neglected Eastern Tianshan Mountains populations.

A robust framework for translating genetic data into conservation action involves defining the Management Unit (MU) [16]. Evolutionarily Significant Unit (ESU) are traditionally defined as populations that exhibit reciprocal monophyly in organellar DNA lineages (e.g., mtDNA or *cp*DNA) and significant divergence in nuclear allele frequencies, reflecting historically isolated lineages with long-term evolutionary independence according to Moritz [8,17,18]. Expanding on this framework, Funk [19] advocate that the ideal delineation of ESU should incorporate evidence of adaptive divergence, rather than relying solely on neutral genetic markers, which may capture historical isolation without necessarily implying adaptive significance. In contrast, MUs are defined on a contemporary timescale based on significant differences in allele frequencies, reflecting restricted gene flow and serving as practical units for immediate management interventions. Integrating these concepts allows for the identification of lineages requiring priority conservation from an evolutionary standpoint while enabling tailored management for specific populations on an ecological timescale.

Advances in molecular biology have profoundly enhanced our ability to delineate such units. Genetic markers have evolved from morphological and biochemical traits to sophisticated molecular tools [20]. Among these, co-dominant microsatellite (SSR) markers are widely used in conservation genetics due to their high polymorphism, reproducibility, and utility in analyzing contemporary genetic diversity and population structure [21]. For example, SSR markers [22] have successfully revealed population differentiation in the endangered Taxus chinensis, informing the delineation of priority conservation units [23]. To complement this contemporary snapshot, sequences from non-coding chloroplast DNA (*cp*DNA) regions (e.g., *trn*L-*trn*F), which are maternally inherited and evolve at a moderate rate [24], are invaluable for reconstructing phylogeographic history and inferring historical population dynamics. A synergistic approach combining fast-evolving SSR markers (reflecting recent demographic processes) with more conserved *cp*DNA and nrDNA sequences (revealing deeper evolutionary history) provides a comprehensive basis for defining independent conservation units.

The primary purpose of this study is to elucidate the genetic relationship between the understudied populations of *S. involucrata* in the Eastern Tianshan Mountains and the populations in the Bayinbuluke area. We employ an integrated molecular approach using eighteen polymorphic SSR markers [15], three *cp*DNA regions (*trn*L-F, *mat*K, *ndh*F-*rpl*32), and the nrDNA ITS1-4 region. Specifically, our objectives are to (1) assess and compare the levels of genetic diversity in Eastern Tianshan Mountains and Bayinbuluke populations; (2) analyze the population genetic structure and differentiation across the study area; (3) assess the relationship between genetic differentiation and geographical environment was inferred; and (4) based on the combined genetic evidence, evaluate the conservation status of the Eastern populations and propose specific protection strategies. The results will provide critical genetic evidence to guide both in situ and ex situ conservation efforts, establishing a solid scientific foundation for the sustainable preservation of this valuable alpine medicinal plant.

## 2. Results

### 2.1. Genetic Diversity

Based on the analysis of genetic diversity parameters in *S. involucrata*, the results are summarized in Table 1. Among the 16 populations examined, the average number of samples per locus per population (*N*) ranged from 3.667 to 20.278, with a mean of 9.660. To comprehensively assess allelic variation, this study analyzed metrics including the mean number of observed alleles (*Na*) and the mean effective number of alleles (*Ne*). *Na* refers to the average number of alleles observed per locus, reflecting allelic richness, whereas *Ne* takes allele frequencies into account, estimating the equivalent number of alleles under equal frequency conditions and thus indicating the evenness of allele distribution. A significant difference between *Na* and *Ne* often suggests the presence of rare alleles or uneven allele frequencies, which may result from genetic drift or recent population bottlenecks. *Na* values ranged from 2.278 to 6.944, and *Ne* ranged from 1.838 to 3.591. The overall means of *Na* and *Ne* across all populations were 3.479 and 2.459, respectively. Population 12 from the Eastern Tianshan Mountains exhibited the highest values for *N*, *Na*, and *Ne*. Observed heterozygosity (*Ho*) across populations varied from 0.306 to 0.534, with an average of 0.402. Expected heterozygosity (*He*) ranged from 0.315 to 0.647, with an overall mean of 0.488. The mean Shannon’s Information Index (*I*) was 0.905, which integrates both allelic richness and evenness and serves as another common indicator of genetic diversity. Regionally, the average genetic diversity indices were higher in populations from the Eastern Tianshan Mountains than in those from the Bayinbuluke area, indicating a relatively higher level of genetic diversity. In contrast, populations 10 and 11 displayed lower values across these metrics, suggesting more limited genetic diversity within these groups.

AMOVA analysis (Table 2) revealed that only 2% of the total genetic variance was attributed to differences among populations, while 60% was found among individuals within populations, and the remaining 38% resided within individuals. This result indicates a low level of genetic differentiation among the studied populations of *S. involucrata*. Overall, the genetic diversity of *S. involucrata* was maintained both within and between the populations of the Eastern Tianshan Mountains and the Bayinbuluke area.

**Figure 1:**
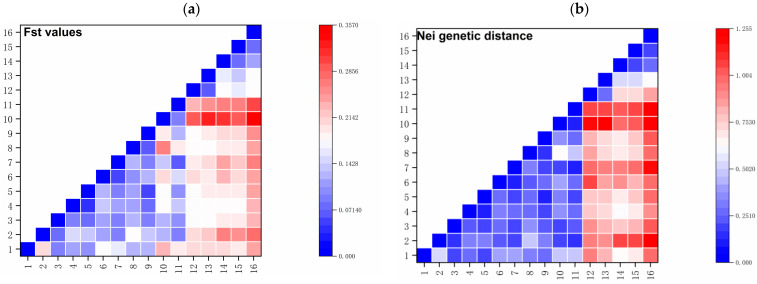
(**a**) Pairwise population matrix of Fst values of *S. involucrata* populations; (**b**) pairwise population matrix of Nei genetic distance of *S. involucrata* populations. 1–11 are populations distributed in the Bayinbuluke area; 12–16 are populations of the Eastern Tianshan Mountains. 12 is a population distributed in the Tianchi area; 13 is a population distributed in the Turpan area; 14–16 are populations distributed in Hami region.

Nei’s genetic distance and pairwise FST values (Table A1 and Table A2) are key parameters for evaluating genetic differentiation among populations (Figure 1). Both metrics are derived from changes in allele frequencies, and they effectively reveal patterns of genetic divergence and relatedness. Analysis of the genetic distance matrix indicates that the distance between population 4 and population 5 was the smallest, reflecting the lowest level of genetic divergence between any two populations. In contrast, the genetic distance between population 10 and population 13 was the largest, indicating the greatest degree of differentiation among all pairwise comparisons. Consistent with this, population 16 exhibited the largest genetic distances when compared to most other populations, suggesting that it is the most genetically distinct group overall. Furthermore, comparisons between populations from the Eastern Tianshan Mountains (populations 12–16) and those from the Bayinbuluke area revealed generally larger genetic distances, highlighting a pattern of genetic differentiation between these two regional groups.

This study assessed recent population bottlenecks in the *S. involucrata* populations by examining the distribution of allele frequencies using the sign test, the Wilcoxon signed-rank test, and a mode-shift indicator. The results revealed distinct distribution patterns among the populations (Table 3). Based on the one-tailed probability for heterozygosity excess under both the Two-Phase Model (T.P.M.) and the Stepwise Mutation Model (S.M.M.), as well as the shape of the allele frequency distribution, the populations were clearly categorized into two groups: those exhibiting a normal L-shaped distribution and those displaying a shifted distribution mode. Analysis indicated that six populations (No. 1, 3, 7, 9, 12, 13) maintained a normal L-shaped distribution, as their test results did not provide significant evidence for a recent bottleneck. Conversely, the allele frequency distribution was shifted in the remaining ten populations (No. 2, 4, 5, 6, 8, 10, 11, 14, 15, 16). Within the latter group, the results further varied. For five populations (No. 2, 11, 14, 15, 16), the sign test and Wilcoxon test *p*-values under both mutation models were greater than 0.05, yet their allele frequency distributions were classified as shifted. A different pattern was observed for four populations (No. 4, 5, 6, 8). For these, the *p*-values for heterozygosity excess under the T.P.M. and S.M.M. were significant (*p* < 0.05), the mode-shift indicator showed a shifted distribution, and while the sign test *p*-value was >0.05, the Wilcoxon test *p*-value was <0.05.

### 2.2. Genetic Structure Analysis

**Figure 2:**
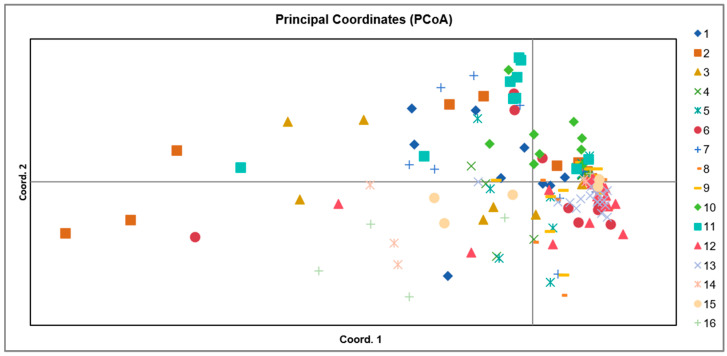
Principal component analysis (PCoA). 1–11 are populations distributed in the Bayinbuluke area; 12 is a population distributed in the Tianchi area; 13 is a population distributed in the Turpan area; 14–16 are populations distributed in the Hami region.

Based on microsatellite data, population genetic structure was investigated using principal coordinate analysis (PCoA; Figure 2) and a Bayesian clustering approach implemented in STRUCTURE. The first three principal coordinates from the PCoA collectively explained 6.24% of the total genetic variation, with individual contributions of 3.17% (PC1), 1.59% (PC2), and 1.46% (PC3). The PCoA plot revealed a generally clustered distribution, with individuals from most populations overlapping broadly in the multivariate space. Although the 16 populations collectively formed one main cluster, a few individuals from several populations were positioned away from their respective group centroids. No clear separation was observed according to geographic source populations, further supporting the presence of extensive gene flow across the sampled range.

**Figure 3:**
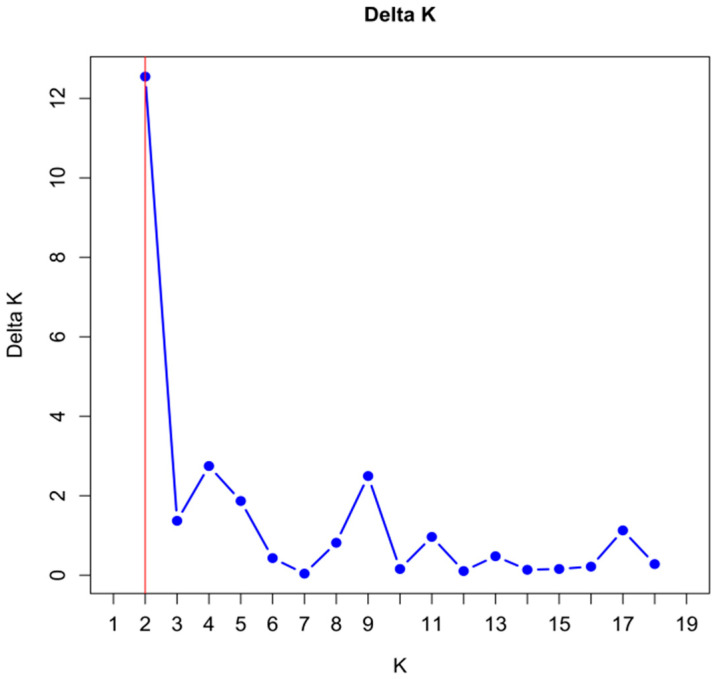
ΔK values with different K parameters, where the optimal number of genetic clusters is determined via the ΔK method. The blue dots show the Delta K values corresponding to different K values. The red vertical line marks the peak of Delta K at K = 2.

**Figure 4:**
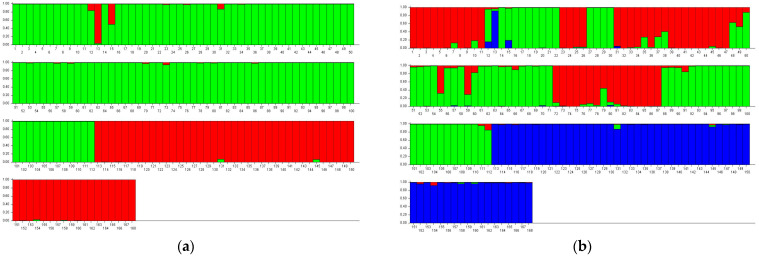
(**a**) ΔK = 2; (**b**) ΔK = 3. Bayesian analysis (STRUCTURE) clustering diagram of *S. involucrata* populations. 1–112 represents populations 1–11, while 113–168 denotes populations 13–16. 1–11 are populations distributed in Bayinbuluke area; 12–16 are populations of Eastern Tianshan Mountains. Each color represents a hypothetical ancestral population, with the proportions reflecting the ancestral composition. The colors themselves have no fixed meaning and are arbitrarily assigned by the software.

To infer population genetic structure, a Bayesian clustering analysis was performed using Structure 2.3.4 software. The ΔK method indicated that the optimal number of genetic clusters (K) was 2 (Figure 3). ΔK = 2, and the 16 populations were clearly divided into two major groups (Figure 4): Group I comprised populations 1–11 from the Bayinbuluke area, and Group II consisted of populations 12–16 from the Eastern Tianshan Mountains. This result reveals that the genetic structure of *S. involucrata* is associated with geographical distribution, reflecting broad-scale divergence between the two regions. At the same time, the individual assignment plots show a degree of shared ancestry or admixture between groups, indicating that historical or ongoing gene flow has occurred across this geographical divide.

**Figure 5:**
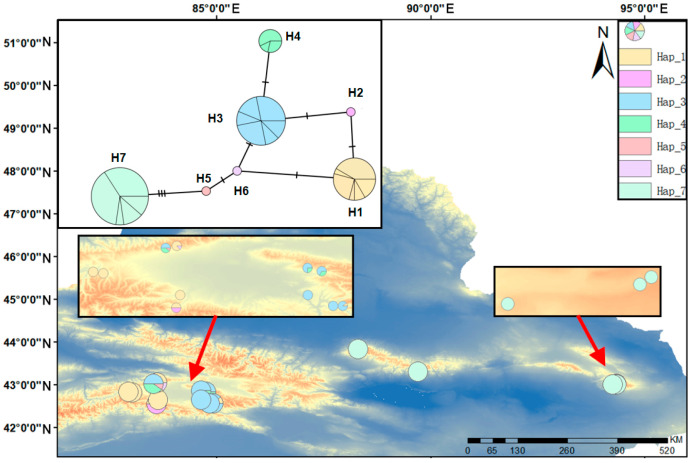
The geographic distributions and frequencies of the nrDNA gene regions detected in the *S. involucrata* populations in the Tianshan Mountains, with their haplotype networks constructed by using Popart-1.7 TCS. The image within the black frame is a close-up view. The red arrow indicates the magnified area.

**Figure 6:**
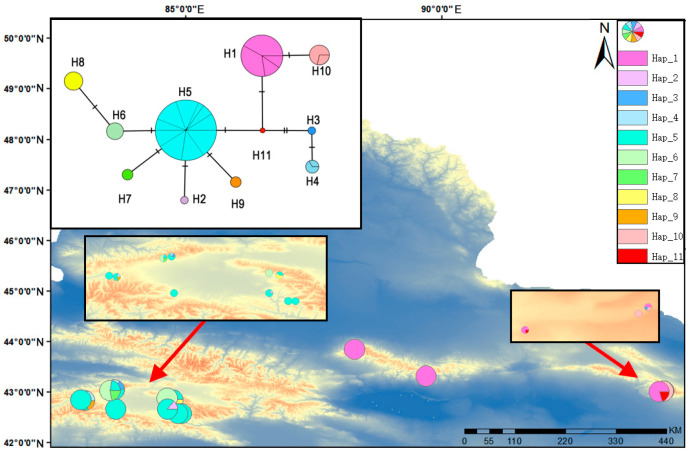
The geographic distributions and frequencies of the *cp*DNA gene regions detected in the *S. involucrata* populations in the Tianshan Mountains, with their haplotype networks constructed by using Popart-1.7 TCS. The image within the black frame is a close-up view. The red arrow indicates the magnified area.

To assess historical phylogeographic patterns, haplotype networks were constructed for both nrDNA (ITS1–4, 558 bp) and *cp*DNA (combined three non-coding regions, 2964 bp) sequences (Figure 5 and Figure 6). A total of 11 *cp*DNA haplotypes (H1–H11) and 7 nrDNA haplotypes (H1–H7) were identified. The analysis revealed distinct geographic structuring. In the *cp*DNA network, several private haplotypes were found in the Eastern Tianshan Mountains populations. While haplotype H5 occupied a central position in the network topology, it was geographically restricted to the Bayinbuluke area. Similarly, haplotype H1 was private to the Eastern Tianshan Mountains populations. This pattern of geographically restricted, private haplotypes suggests a history of prolonged isolation and independent evolutionary trajectories between the regions. The presence of shared haplotype H3 may indicate historical gene flow or retention of ancestral polymorphism. The nrDNA haplotype distribution was consistent with this pattern, with haplotype H7 being exclusive to the Eastern Tianshan Mountains. Overall, haplotype richness was markedly higher in the Bayinbuluke area, where the widespread haplotype H3 was also the most frequent. The combined evidence from both markers demonstrates significant differences in *cp*DNA and nrDNA haplotype composition between the Eastern Tianshan Mountains populations and the Bayinbuluke region. The network structure, with central haplotype H5 giving rise to the private Eastern Tianshan Mountains haplotype H1, supports a scenario of genetic divergence following long-term geographical isolation. The shared occurrence of haplotype H3 likely reflects either ancestral shared ancestry or limited historical gene exchange. The present genetic structure confirms that populations from the two regions have undergone significant genetic differentiation.

**Figure 7:**
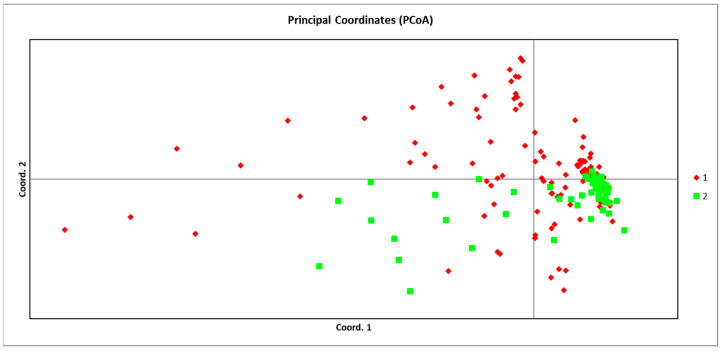
Principal component analysis (PCoA). 1 represents the Bayinbuluke area and 2 represents the Eastern Tianshan Mountains.

To further evaluate the regional genetic structure, populations from the Eastern Tianshan Mountains and the Bayinbuluke area were grouped and subjected to principal coordinate analysis (PCoA) (Figure 7). The results revealed a clear spatial separation along the primary axes. Populations from the Bayinbuluke area exhibited a broad distribution in the PCoA plot, indicating higher genetic variation within this region. In contrast, populations from the Eastern Tianshan Mountains clustered more tightly and were positioned distinctly below those from Bayinbuluke along the major axis of variation. This spatial segregation in the PCoA provides independent support for the genetic structure inferred from both the SSR marker analysis and the sequence-based (*cp*DNA and nrDNA) haplotype networks. Collectively, all lines of evidence confirm that *S. involucrata* populations from the Eastern Tianshan Mountains and the Bayinbuluke area possess a significant and reproducible genetic structure.

**Figure 8:**
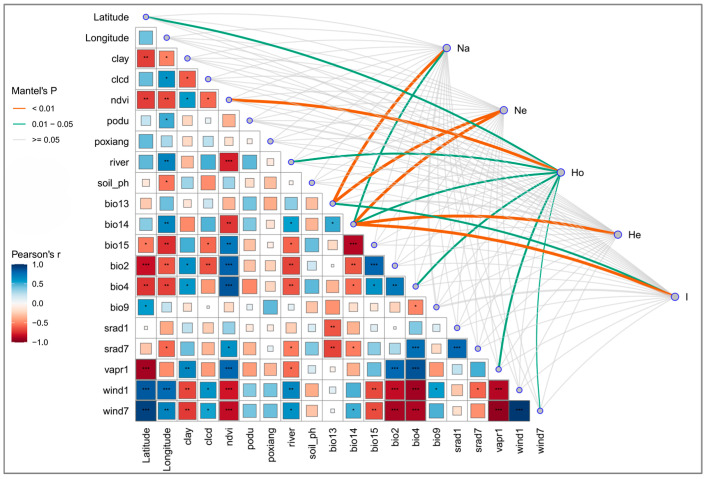
Mantel test of *S. involucrata* based on SSR molecular markers. An asterisk (*) indicates statistical significance: * represents statistical significance at the 95% confidence level (*p*-value < 0.05), ** at the 99% confidence level (*p*-value < 0.01), and *** at the 99.9% confidence level (*p*-value < 0.001).

To investigate the association between environmental factors and genetic diversity in *S. involucrata*, this study employed Mantel tests to assess the correlation between genetic differentiation and geographic–environmental variables, to explore evolutionary hypotheses, and to analyze multivariate data. The Mantel test results indicated that the genetic diversity indices derived from SSR markers in *S. involucrata* were correlated with environmental factors. A significant correlation was observed between observed heterozygosity (*Ho*) and the Normalized Difference Vegetation Index (NDVI), which reveals a direct link between the genetic diversity of *S. involucrata* and the productivity and health status of its ecosystem. The study also found significant correlations between the mean number of alleles (*Na*) and the effective number of alleles (*Ne*) with precipitation of the wettest month (Bio13). Additionally, precipitation of the driest month (Bio14) showed significant correlations (*p* < 0.01) with *Ne*, expected heterozygosity (*He*), and Shannon’s Information Index (*I*) (Figure 8).

**Figure 9:**
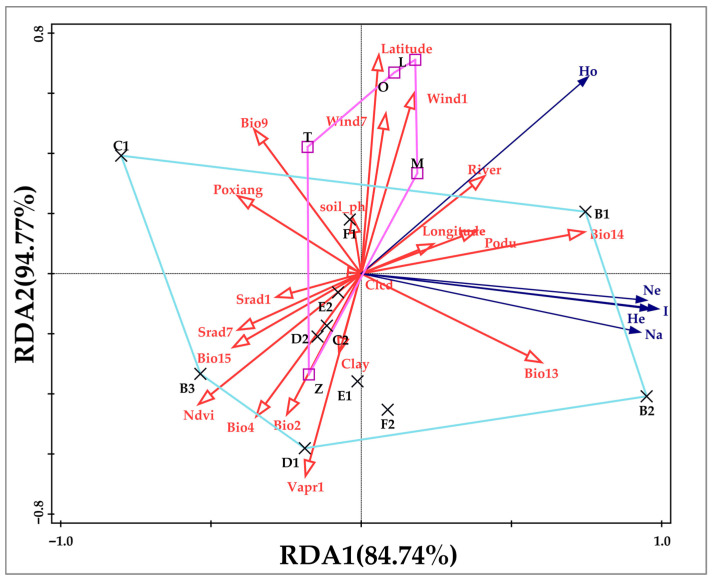
Redundancy analysis of *S. involucrata* based on SSR molecular markers. On the horizontal axis, 84.74% of the data variance is explained, while on the vertical axis, 94.77% of the variance is revealed. Red represents geographical environmental factors. Black represents the population number. Blue represents genetic diversity parameters.

To investigate both direct and indirect relationships among the variables, redundancy analysis (RDA) was further employed to explore the associations between genetic variation and specific environmental factors. The RDA results indicated that precipitation (Bio13 and Bio14) had a highly significant effect on the genetic variation of *S. involucrata*, which is consistent with the findings from the Mantel tests. The analysis also revealed that longitude, slope (Podu), and river proximity exerted influences on genetic variation (Figure 9).

## 3. Discussion

Human activities are recognized as a primary driver of species population decline and extinction [25]. For many species, anthropogenic pressures such as habitat fragmentation and overexploitation have precipitated sharp reductions in wild population sizes, often confining remaining individuals to small, isolated fragments. This demographic trajectory frequently triggers a cascade of negative genetic consequences, including the erosion of genetic diversity, increased inbreeding depression, and the intensification of genetic drift, which collectively elevate extinction risk [26,27]. This study employed a combination of SSR markers and Sanger sequencing of *cp*DNA and nrDNA regions to systematically evaluate the genetic diversity and population structure of *S. involucrata* in the Eastern Tianshan Mountains. In our study, we analyzed the association between population genetic differentiation and environmental factors in *S. involucrata*. Our integrated analysis reveals a distinct genetic signature for these eastern populations. Therefore, based on the cumulative genetic evidence presented here, we propose that the populations in the Eastern Tianshan Mountains should be delineated and managed as an independent Management Unit.

### 3.1. Genetic Diversity of S. involucrata in Tianshan Mountains

Analysis of molecular variance (AMOVA) and population genetic parameters indicate that the majority of genetic variation in *S. involucrata* resides among and within individuals, with only minimal differentiation observed between populations. This pattern suggests historically frequent gene flow across its range. The average expected heterozygosity (*He*) was 0.557 in the Eastern Tianshan Mountains populations and 0.457 in the Bayinbuluke area populations. These values are comparatively high for an alpine specialist; for instance, the congeneric *Saussurea medusa* exhibits a species-level *He* of only 0.276 [28]. This relatively high diversity suggests that *S. involucrata* may not have undergone severe historical genetic bottlenecks, possibly due to persistence in regional refugia during past glacial cycles [29].

However, genetic diversity is not uniformly distributed across the landscape. Our results demonstrate that populations in the Eastern Tianshan Mountains harbor particularly high genetic diversity, which may reflect stronger genetic adaptability. This disparity could be explained if the Eastern Tianshan Mountains acted as a major glacial refugium for the species, allowing for the accumulation and preservation of ancestral genetic variation. The long-term geographical isolation between the Eastern Tianshan and Bayinbuluke populations, potentially driven by pronounced climatic gradients, may have further promoted differentiation. As noted by Hu [14], the Western Tianshan and Altai Mountains receive greater moisture via westerly airflows, whereas the Eastern Tianshan is comparatively arid. This proposed link between genetic patterns and environmental drivers, specifically precipitation, is robustly supported by our direct statistical assessments. To explicitly investigate the association between environmental factors and genetic diversity, this study employed Mantel tests and redundancy analysis (RDA). The Mantel tests confirmed significant correlations between multiple genetic diversity indices (e.g., *Ne*, *He*, *I*) and key precipitation variables, including precipitation of the driest month (Bio14). Furthermore, the significant correlation between observed heterozygosity (*Ho*) and the Normalized Difference Vegetation Index (NDVI) reveals a direct link between genetic diversity and ecosystem productivity. The RDA further substantiated that precipitation exerts a highly significant effect on genetic variation, consistent with the Mantel results, and also highlighted influences of longitude, slope, and river proximity. These findings collectively confirm that the relatively high genetic diversity in the Eastern Tianshan region may reflect adaptability to this harsher environment, as well as to the potentially more complex habitat types found there. Habitat heterogeneity is a key driver for maintaining genetic diversity, as varied environmental conditions can select for different adaptive genotypes, thereby preserving a broader genetic base.

Analysis of recent demographic history using bottleneck detection methods revealed that Eastern Tianshan populations 12 and 13 exhibit a characteristic L-shaped distribution of allele frequencies [30,31]. This pattern provides strong evidence that these populations have maintained relatively large and stable effective population sizes over time, with minimal impacts from genetic drift. The absence of a genetic bottleneck signature in these groups is particularly significant, as it indicates an enhanced capacity to retain low-frequency alleles and a high degree of genetic resilience. Consequently, populations 12 and 13 should be considered vital reservoirs of the species and genetic diversity and prioritized as core source populations in conservation strategies. In contrast, the shifted allele distribution observed in three other Eastern Tianshan Mountains populations (Nos. 14, 15, 16) should be interpreted with caution. These results may arise from statistical artifacts or sampling error rather than genuine demographic decline [32]. Small sample sizes can reduce the accuracy of allele frequency estimation, potentially leading to false signals of a bottleneck. Further sampling would be needed to clarify the demographic history of these particular groups.

### 3.2. Populations’ Genetic Structure and Evolutionary History

A deeper level of genetic structure was revealed through principal coordinate analysis (PCoA), Bayesian clustering, and haplotype network reconstruction. In the principal coordinate analysis (PCoA), the population of the Eastern Tianshan Mountains forms an independent cluster, which is located below the cluster in the Bayinbulak area. This spatial segregation indicates that genetic variation within the Eastern Tianshan Mountains populations is structured and differentiated from the Bayanbulak area. Simultaneously, our findings also indicate that longitude, slope, and river proximity have an influence on genetic variation. Therefore, we propose that this genetic differentiation likely arose from a combination of topographic complexity and environmental–climatic factors. This pattern suggests that the Eastern Tianshan Mountains population lineage may represent a locally specialized genetic group, potentially derived from an ancestral population that originally differentiated in the Western Tianshan Mountains region. Such heterogeneous genetic structuring is common in narrowly distributed alpine plants and is often associated with shifts in flowering phenology driven by temperature gradients, habitat fragmentation imposed by complex mountain terrain, and naturally limited gene flow [33]. The major geological history of the region provides a critical backdrop for interpreting these patterns. The multi-stage deformation in the Tianshan area is widespread in the Qinghai–Tibet Plateau and its surrounding areas. It is likely to be a synchronous response to the multi-stage uplift process of the Qinghai–Tibet Plateau [34]. This uplift not only reshaped the topography of the Tianshan but also intensified aridity in the surrounding regions [35]. Such profound geological events have had lasting impacts on the genetic architecture of regional flora [36]. Furthermore, habitat fragmentation, a consequence of such topographic changes, can reduce within-population genetic diversity while amplifying genetic differences between populations [37]. The increased aridity in the Eastern Tianshan Mountains populations compared to the west, itself influenced by plateau uplift, may be a key driver shaping the observed patterns of genetic diversity and structure in *S. involucrata*.

Importantly, the *cp*DNA and nrDNA haplotype analyses provide direct evidence for this evolutionary history. The presence of private haplotypes in the Eastern Tianshan Mountains (H1 in *cp*DNA and H7 in nrDNA) highlights their genetic distinctiveness. The shared *cp*DNA haplotype H3 may indicate either limited historical gene exchange or the retention of an ancestral polymorphism following population subdivision. The contrasting spatial patterns of *cp*DNA and nrDNA lineages suggest that the Tianshan populations have experienced prolonged geographical isolation leading to independent evolution. The mountains and valleys of the region likely act as effective barriers to gene flow. It is noteworthy that *cp*DNA primarily captures recent, localized maternal expansion events mediated by seed dispersal, whereas nrDNA more strongly reflects deeper phylogenetic structure shaped by long-term geographic isolation [38]. Therefore, this study infers infer that long-term, though not complete, geographical isolation exists between the Eastern Tianshan Mountains populations and Bayinbuluke populations, resulting in restricted gene flow. This isolation can be attributed to the limited seed dispersal capacity of *S. involucrata* [39] coupled with the significant geographic barriers presented by the terrain separating the two regions [40]. Together, these factors have likely enabled the formation and maintenance of the observed population genetic structure.

### 3.3. Association Between Genetic Patterns and Reproductive Ecology

The genetic patterns revealed by *cp*DNA and nuclear markers can be explained by the reproductive biology of *S. involucrata*. This species is a perennial, insect-pollinated, hermaphroditic (perfect-flowered) herb with a predominantly facultative outcrossing mating system. Although self-compatible, it relies heavily on pollinators for effective pollination [41]. Gene flow in *S. involucrata* is mediated by both seeds and pollen, which differentially shape the maternal *cp*DNA and nrDNA markers, resulting in distinct marker-dependent genetic structures.

In angiosperms, *cp*DNA is typically strictly maternally inherited; its haplotype distribution directly records the history of seed dispersal and maternal lineage migration. The pronounced east–west *cp*DNA phylogeographic break observed in our study, along with private haplotypes in the eastern populations, indicates limited seed-mediated gene flow over evolutionary timescales. This aligns with the species’ reproductive ecology. Although the seeds have crown hairs, successful seedling establishment in harsh, fragmented alpine scree habitats is extremely low [42,43]. Long-distance colonization across vast unsuitable terrain is even more constrained. Thus, the strong *cp*DNA divergence likely reflects historical isolation among glacial refugia, followed by independent evolution of the Eastern Tianshan populations’ maternal lineages driven largely by genetic drift under restricted seed dispersal.

In contrast, the shallow population differentiation and admixed signals revealed by SSR markers suggest that pollen-mediated gene flow—though limited—acts as a more effective homogenizing force than seed dispersal. *Bombus* spp., *Calliphora uralensis*, *Pontia callidice*, *Clossiana euphrosane*, and *Aglais urticae* are the primary pollinating insects of *S. involucrata* [41]. These pollinators facilitate pollen transfer at local to regional scales, partly counteracting genetic differentiation. However, their movement is strongly constrained by the complex topography and climate of the Tianshan Mountains, leading to an isolation-by-resistance pattern. Pollen flow declines not only with distance but is also impeded by deep valleys, ridges, and extensive unsuitable habitat between eastern and western regions. Consequently, the observed nuclear genetic structure reflects a dynamic balance among historical isolation, contemporary restricted pollen flow, and genetic drift. In summary, the genetic architecture of *S. involucrata* may result from the interplay between its reproductive traits and geographical setting. Restricted seed dispersal has led to strong a phylogeographic structure in *cp*DNA, while limited but ongoing pollen flow has buffered genetic differentiation in the genome.

### 3.4. Management Unit Division

In the integrated analysis, the congruent patterns of significant genetic differentiation observed in *cp*DNA and nrDNA sequence data are further corroborated by the independent SSR analysis. However, the data support that the sampled populations are independent Management Units, but do not meet the ESU conceptual standard proposed by Funk (Evolutionarily Significant Unit: a group of conspecific populations that has substantial reproductive isolation, which has led to adaptive differences so that the populations represent a significant evolutionary component of the species) [19]. The nuclear differentiation and admixture observed in our SSR and Sanger sequencing data are more consistent with the criteria for a Management Unit (MU). Our study data indicate that although the Eastern Tianshan populations possess private *cp*DNA and nrDNA haplotypes and exhibit significant differences in genetic diversity compared to the Bayanbulak area (significant FST and clear clustering into two groups in STRUCTURE analysis), they lack reciprocal monophyly in *cp*DNA, exhibit relatively shallow nuclear genome differentiation (SSR shows obvious admixture and PCoA overlap), and fail to provide evidence for reproductive isolation. Therefore, it does not meet the criteria for ESU designation in any respect. Meanwhile, the observed allele frequency differences, private alleles and haplotypes, and genetic structure under geographic isolation fully satisfy the criteria for MU designation. This consistency across multiple genetic datasets underscores a distinct, independent evolutionary history for the Eastern Tianshan Mountains populations’ lineage. Complementing this evolutionary perspective, the contemporary genetic structure inferred from SSR markers and the Bayesian clustering analysis (STRUCTURE) provides the basis for practical conservation management. We therefore propose the establishment of a distinct Management Unit (MU) for the Eastern Tianshan Mountains populations. This unit, characterized by its high genetic diversity, represents a crucial reservoir of adaptive potential for the species and should be the focus of targeted conservation strategies.

### 3.5. Protection Recommendations

This study posits that the Eastern Tianshan Mountains populations should be managed as an independent Management Unit. It is the main measure to protect the evolutionary trajectory of the species. Priority protection should be given to populations 12 and 13 with high genetic diversity and special haplotype populations. At present, the focus of protection is to implement preventive in situ protection, strictly maintain the existing population size and habitat integrity, and focus on protecting the unique *cp*DNA haplotypes to prevent genetic erosion. In order to fully maintain the adaptive evolutionary potential of *S. involucrata* in the Eastern Tianshan Mountains, it is necessary to implement a comprehensive protection strategy covering the entire distribution area: (1) Seed bank construction. The collection of germplasm resources should systematically cover the eastern part of the Tianshan Mountains to ensure the representativeness of all evolutionary lineages of the species. The germplasm resources in this area should be systematically collected, and a safe ecological backup should be established. (2) For the Management Unit of Tianshan Mountains, a protection strategy with habitat protection and genetic monitoring as the core should be implemented to prevent the decline in its diversity. It is recommended to establish permanent genetic monitoring plots to regularly evaluate the genetic diversity, inbreeding level, and gene flow changes of key populations, enabling us to scientifically evaluate the effect of protection measures and dynamically adjust the management plan. (3) We suggest strengthening the efficiency of law enforcement preventing illegal collection. Standardizing tourism and animal husbandry activities and minimize the interference of trampling and overeating are also recommened. (4) By transforming local herdsmen into ecological guardians and integrating them into the main body of conservation action, the establishment of community-based collaborative management mechanisms can be promoted. (5) At the same time, in order to realize the overall protection and sustainable protection of the genetic diversity of *S. involucrate*, we need to establish a collaborative protection mechanism for *S. involucrata* throughout the Tianshan Mountains.

## 4. Materials and Methods

All plant materials used in this study were collected from fresh leaf samples of the endangered species *S. involucrata* obtained from its natural populations in Xinjiang, China. A total of 168 individual specimens were sampled from 16 geographically distinct populations. Detailed locality information for each population is provided in Table 4. Given that this species is rare, with a fragmented habitat and a highly restricted distribution, conducting field surveys and collecting samples posed significant challenges. Therefore, our sampling protocol was designed to obtain a genetically representative snapshot of the existing populations while minimizing ecological disturbance. This principle guided our overall strategy for determining inter-plant spacing and sample size. To ensure genetic independence among samples and reduce potential biases from spatial autocorrelation or clonal reproduction, a minimum distance of at least 10 m was maintained between any two sampled individuals within each population. This measure helped lower the likelihood of collecting genetically identical individuals. When a suspected clonal cluster was encountered, only one representative individual was sampled from it. To ensure that the sampled units represented distinct population units, a minimum distance of >5 km was maintained between the sampling sites of different populations. The sample size per population (*n* = 5–21 individuals) was determined based on factors such as the endangered status of the plants and the difficulty of sampling. Fresh leaf tissue was collected from each selected plant, immediately dried in silica gel, and stored at −20 °C. DNA was subsequently extracted from the silica-dried leaves using a modified CTAB method. Key environmental parameters were recorded at each sampling site.

Amplification using SSR markers was conducted with 18 polymorphic primer pairs that produced clear and stable amplification, designed in accordance with Hu [15]. Additionally, data from Hu’s study, comprising 112 individuals across 11 populations distributed in the Bayinbuluke region, were utilized. The PCR amplification reaction system had a total volume of 25 μL, which included 1 μL of template DNA, 1 μL of upstream primer, 1 μL of downstream primer, 12.5 μL of 2× Easy Taq PCR Super Max, and 9.5 μL of ddH_2_O. The amplification method included three stages: initial pre-denaturation at 93 °C for 3 min, followed by 35 cycles of denaturation at 94 °C for 30 s and annealing at 54 °C for 30 s (adjusted according to the specific Tm value of each primer combination), followed by an extension step at 65 °C for 90 s, and a final extension at 65 °C for 5 min. Successful amplification was confirmed by 2% agarose gel electrophoresis. PCR products were identified through capillary electrophoresis and fluorescent labeling. The amplification reaction system and methods were conducted in accordance with Hu [15]. Sanger sequencing was conducted on three regions of chloroplast DNA (*trn*L-*trn*F, *mat*K, and *ndh*F-*rpl*32). Primers were obtained from [13,14]. The ITS1-4 locus of the nuclear genome was amplified. The amplification reaction mixture and thermal cycling protocol were used in accordance with Shi [13].

For SSR data tesults, GenAlEx 6.51b2 [44] was employed to identify the populations’ genetic parameters for each primer pair, including the number of alleles (*Na*), observed heterozygosity (*Ho*), expected heterozygosity (*He*), and effective number of alleles (*Ne*) [45]. Principal component analysis (PCoA) and AMOVA were also conducted using GenAlEx 6.51b2. Bayesian analysis was performed using Structure 2.3.4 software [46] to determine the optimal number of genetic clusters (K) for all populations. This study employed the Admixture model, which allows individual genomes to originate from multiple ancestral populations, as it is suitable for scenarios involving gene flow or recent population admixture. To assist with clustering under weak population structure, this study employed the LOCPRIOR model, incorporating the sampling location information of individuals as prior data. The parameters were set as follows: 10,000 iterations for the Length of the Burn-in Period and 100,000 iterations for the Number of MCMC Replications after Burn-in. The K-value was tested from 1 to 19, with 20 independent runs for each K-value. The results from all runs were then compressed and imported into the online platform Structure Harvester for analysis to determine the most suitable K-value. Finally, the Structure 2.3.4 clustering analysis plot was generated. We used Bottleneck v. 1.2.02 [31,47] to evaluate whether the *S. involucrata* populations have recently experienced a bottleneck event. The computational models used were the Stepwise Mutation Model (SMM) and the Two-Phase Mutation Model (TPM), both developed based on the number of alleles. The TPM, which is more suitable for SSR data analysis, was configured with the following parameters: SSM at 90%, a mutation rate of 30%, and 1 × 10^3^ repetitions. The sign test and Wilcoxon signed-rank test were employed on the 11 populations to evaluate whether excess heterozygosity was statistically significant within each population [48]. This study employed Origin 2024 software to create genetic diversity index maps for the populations of the Eastern Tianshan Mountains and Western Tianshan Mountains, using box plots. The Nei genetic distance and *Fst* value matrix among populations were computed using GenAlEx 6.51b2 software. The resulting matrix data were imported into Origin 2024 software to produce the basic heat map, modify the scale based on the data range, activate populations labels and numerical annotations, and refine the settings of the coordinate axes and grid lines.

The obtained DNA sequences were imported into Geneious Prime 2024.0.5 [49] for quality assessment and assembly. Following visual inspection of chromatograms to confirm base calls, sequences were manually trimmed to remove low-quality ends and vector contamination. Overlapping sequence fragments were then assembled to generate consensus sequences for each individual. Haplotype identification, along with calculations of haplotype diversity (Hd) and nucleotide diversity (π), was performed using DnaSP v. 5 [50]. The sampling points and their corresponding haplotype data were mapped using ArcMap 9.3 software to generate a haplotype geographic distribution map. Finally, Popart-1.7 [51] was used to construct a median-joining haplotype network, which visualizes the phylogenetic relationships among haplotypes.

This study considered 20 environmental factors (Table A3 and Table A4). Climatic factor data were derived from the WorldClim database [52], while topographic factors such as slope and aspect were calculated from 30 m resolution Digital Elevation Model (DEM) data obtained from the Geospatial Data Cloud platform. The distance to rivers was computed as the Euclidean distance based on the 1:1,000,000 national river dataset downloaded from the Geospatial Data Cloud. The China Annual Maximum Normalized Difference Vegetation Index (NDVI) dataset, with a spatial resolution of 30 m and spanning 2000–2020, was provided by the Land Use and Global Change Remote Sensing Team of the Institute of Geographic Sciences and Natural Resources Research, Chinese Academy of Sciences. To evaluate the relationship between genetic differentiation and geographic–environmental variables [53], this study employed an integrated analytical framework combining Mantel tests and redundancy analysis (RDA) based on microsatellite (SSR) markers. Geographic (Euclidean) distance and environmental distance matrices were generated from standardized bioclimatic variables as well as longitude and latitude data. Mantel tests were performed using the R package linkET [54] with 9999 permutation tests to examine the correlations between the genetic distance matrix and each environmental distance matrix. The results were visualized using ggplot2 [55], where edge width corresponds to the Mantel’s r value [56] and color indicates statistical significance. Subsequently, redundancy analysis was conducted using the vegan package to investigate the multivariate influence of environmental factors on within-population genetic diversity indices, including observed heterozygosity, expected heterozygosity, mean number of alleles, effective number of alleles, and Shannon’s Information Index. Key environmental predictors were identified through forward selection (α = 0.05). Finally, quadratic regression models were fitted to characterize nonlinear relationships between the significant environmental variables identified by RDA and each genetic diversity index, thereby elucidating the relative contribution of environmental factors to the genetic diversity pattern in *S. involucrata.* All analyses were conducted in the R statistical environment [57].

To delineate conservation units, SSR molecular markers were combined with Sanger sequencing data to methodically identify key evolutionary units and Management Units. In this approach [17,18], Evolutionarily Significant Unit (ESU) [58] are defined based on pronounced phylogeographic differentiation, reflecting prolonged evolutionary isolation. Subsequently, within each MU, significant differences in allele frequencies among populations were evaluated using SSR data indicative of current gene flow, complemented by Bayesian analysis [16]. Independent Management Units (MUs) with limited gene flow were identified. This systematic approach ensures that the defined units accurately represent the distinctiveness of deep evolutionary history while also informing conservation management focused on current populations.

## 5. Conclusions

This study clarifies the genetic relationship between *S. involucrata* populations in the Eastern Tianshan Mountains and those in the Bayinbuluke region. It further identifies key factors shaping the genetic structure of the Eastern Tianshan populations and proposes targeted conservation measures. Our results indicate that the Eastern Tianshan populations maintain significantly higher genetic diversity than those in Bayinbuluke. More importantly, they form a distinct genetic cluster characterized by private haplotypes, reflecting a history of prolonged geographical isolation. This clear genetic demarcation supports an independent evolutionary trajectory for the eastern lineage. The topographic complexity of the Tianshan range, combined with the distinctive environmental and climatic conditions of the eastern region, appears to have played a key role in driving this genetic differentiation. These heterogeneous abiotic factors likely interact to shape gene flow, influence genetic drift, and promote local adaptation, collectively structuring the population genetics of *S. involucrata*.

Analysis of the relationship between genetic diversity and environmental factors provides direct evidence for these drivers. Multiple genetic diversity indices, particularly parameters related to heterozygosity and allelic richness, showed significant correlations with key environmental variables such as vegetation index (NDVI) and precipitation. Redundancy analysis (RDA) further confirmed that precipitation is the dominant environmental factor driving genetic variation, with topographic and geographic factors also exerting significant influence.

Given the high genetic diversity and observed evidence of adaptive differentiation in the Eastern Tianshan populations, this study suggests that this region be designated as an independent Management Unit (MU). Prioritizing the conservation of this unique genetic diversity is crucial to prevent its erosion due to ongoing habitat fragmentation. In addition to in situ protection, we recommend establishing a germplasm bank to serve as an ecological safety backup for these genetic resources. In summary, this study provides critical genetic evidence to inform and refine conservation strategies, highlighting the urgent need to protect the unique evolutionary potential and adaptive genetic variation of this endangered alpine species. Future research incorporating genomic approaches and ecological niche modeling is recommended to further elucidate the molecular basis of local adaptation.

## Figures and Tables

**Table 1:** Genetic diversity parameters of 16 populations.

Pop *	*N* **	*Na*	*Ne*	*I*	*Ho*	*He*
1	10.056	3.056	2.370	0.802	0.342	0.457
2	7.444	2.889	2.242	0.767	0.422	0.440
3	8.667	3.556	2.575	0.933	0.359	0.518
4	9.556	2.944	2.308	0.855	0.409	0.505
5	9.444	2.944	2.314	0.851	0.378	0.501
6	9.111	3.222	2.668	0.858	0.357	0.477
7	9.167	3.167	2.230	0.830	0.358	0.472
8	9.778	3.000	2.301	0.824	0.316	0.481
9	10.667	3.278	2.271	0.841	0.366	0.467
10	9.556	2.278	1.838	0.537	0.349	0.315
11	8.944	2.667	1.954	0.680	0.306	0.396
12	20.278	6.944	3.591	1.377	0.481	0.647
13	19.611	6.222	3.005	1.217	0.534	0.598
14	4.167	3.389	2.719	0.929	0.459	0.499
15	4.444	3.111	2.442	0.930	0.503	0.537
16	3.667	3.000	2.525	0.880	0.488	0.503
Total						
Mean	9.660	3.479	2.459	0.882	0.402	0.488

* 1–11 are populations distributed in the Bayinbuluke area; 12–16 are populations of the Eastern Tianshan Mountains. 12 is a population distributed in the Tianchi area; 13 is a population distributed in the Turpan area; 14–16 are populations distributed in the Hami region. ** Number of alleles (*N*); effective number of alleles (*Na*); effective number of alleles (*Ne*); Shannon diversity index (*I*); observed heterozygosity (*Ho*); expected heterozygosity (*He*).

**Table 2:** Analysis of molecular variance within and among populations.

Source	df	SS	MS	Est. Var.	%
Among Pops	15	278.385	18.559	0.221	2%
Among Indiv	152	2124.266	13.975	5.317	60%
Within Indiv	168	561.500	3.342	3.342	38%
Total	335	2964.152		8.880	100%

**Table 3:** Detection of bottleneck effect in *S. involucrata* populations.

Pop *	Sign Test		Wilcoxon Test		Mode-Shift
			One Tail for H Excess		
	T.P.M	S.M.M	T.P.M	S.M.M	
1	0.10949	0.1173	0.00381	0.00775	normal L-shaped distribution
2	0.23669	0.43235	0.1156	0.18773	shifted mode
3	0.57317	0.41447	0.20188	0.33885	normal L-shaped distribution
4	0.00934	0.0097	0.00775	0.00912	shifted mode
5	0.04297	0.04552	0.00912	0.00912	shifted mode
6	0.01408	0.01491	0.00623	0.01077	shifted mode
7	0.52273	0.50003	0.37333	0.48161	normal L-shaped distribution
8	0.01852	0.02227	0.00467	0.01008	shifted mode
9	0.43579	0.42229	0.44498	0.5	normal L-shaped distribution
10	0.2138	0.2304	0.03369	0.06152	shifted mode
11	0.14523	0.3319	0.2166	0.24771	shifted mode
12	0	0	0.9972	0.99974	normal L-shaped distribution
13	0.00671	0.0015	0.99763	0.99974	normal L-shaped distribution
14	0.54465	0.53977	0.57654	0.59802	shifted mode
15	0.48775	0.5042	0.41586	0.44929	shifted mode
16	0.18661	0.19358	0.03696	0.04672	shifted mode

* 1–11 are populations distributed in the Bayinbuluke area; 12–16 are populations of the Eastern Tianshan Mountains. 12 is a population distributed in the Tianchi area; 13 is a population distributed in the Turpan area; 14–16 are populations distributed in the Hami region.

**Table 4:** Information of 16 populations.

Number *	Pop	Latitude	Longitude
1	B1 (11) **	43.03° N	83.50° E
2	B2 (11)	43.04° N	83.59° E
3	B3 (9)	42.65° N	84.62° E
4	C1–C10 (10)	42.57° N	84.82° E
5	C11–C20 (10)	42.57° N	84.90° E
6	D1–D10 (10)	42.84° N	82.92° E
7	D11–D20 (10)	42.82° N	83.01° E
8	E1–E10 (10)	42.87° N	84.62° E
9	E11–E21 (11)	42.84° N	84.73° E
10	F1–F10 (10)	42.65° N	83.61° E
11	F11–F20 (10)	42.55° N	83.58° E
12	Z1–Z20 (21)	43.84° N	88.29° E
13	T1–T20 (20)	43.31° N	89.68° E
14	L1–L5 (5)	43.01° N	94.33° E
15	M1–M5 (5)	43.02° N	94.34° E
16	O1–O5 (5)	43.00° N	94.25° E

* 1–11 are populations distributed in the Bayinbuluke area; 12–16 are populations of the Eastern Tianshan Mountains. 12 is a population distributed in the Tianchi area; 13 is a population distributed in the Turpan area; 14–16 are populations distributed in the Hami region. ** The number in parentheses is the sample size.

## Data Availability

The data presented in this study are available on request from the corresponding author.

## Appendix A

**Table A1:** Pairwise Fst values of *S. involucrata* populations.

Pop *	1	2	3	4	5	6	7	8	9	10	11	12	13	14	15	16
1	0.000															
2	0.211	0.000														
3	0.094	0.074	0.000													
4	0.115	0.147	0.095	0.000												
5	0.095	0.139	0.073	0.037	0.000											
6	0.171	0.113	0.116	0.137	0.129	0.000										
7	0.159	0.087	0.088	0.118	0.092	0.058	0.000									
8	0.127	0.186	0.128	0.113	0.110	0.106	0.126	0.000								
9	0.127	0.143	0.088	0.083	0.055	0.122	0.095	0.067	0.000							
10	0.230	0.128	0.128	0.189	0.170	0.213	0.134	0.271	0.200	0.000						
11	0.199	0.086	0.106	0.132	0.099	0.147	0.067	0.202	0.126	0.086	0.000					
12	0.206	0.203	0.175	0.167	0.170	0.209	0.199	0.183	0.184	0.289	0.229	0.000				
13	0.211	0.220	0.189	0.183	0.193	0.200	0.213	0.181	0.193	0.325	0.255	0.066	0.000			
14	0.203	0.271	0.202	0.180	0.199	0.234	0.246	0.202	0.203	0.321	0.273	0.170	0.155	0.000		
15	0.191	0.252	0.192	0.180	0.195	0.204	0.225	0.191	0.206	0.295	0.265	0.157	0.135	0.074	0.000	
16	0.244	0.285	0.233	0.229	0.249	0.244	0.273	0.243	0.259	0.356	0.302	0.188	0.167	0.108	0.079	0.000

* 1–11 are populations distributed in the Bayinbuluke area; 12 is a population distributed in the Tianchi area; 13 is a population distributed in the Turpan area; 14–16 are populations distributed in the Hami region.

**Table A2:** Pairwise matrix of Nei genetic distance of *S. involucrata* populations.

Pop *	1	2	3	4	5	6	7	8	9	10	11	12	13	14	15	16
1	0.000															
2	0.488	0.000														
3	0.139	0.091	0.000													
4	0.200	0.267	0.169	0.000												
5	0.149	0.249	0.108	0.026	0.000											
6	0.368	0.176	0.205	0.247	0.226	0.000										
7	0.331	0.113	0.126	0.198	0.125	0.066	0.000									
8	0.260	0.422	0.291	0.209	0.206	0.188	0.261	0.000								
9	0.240	0.264	0.171	0.134	0.063	0.231	0.145	0.088	0.000							
10	0.442	0.141	0.154	0.315	0.262	0.360	0.158	0.591	0.331	0.000						
11	0.411	0.086	0.156	0.217	0.120	0.231	0.064	0.430	0.204	0.058	0.000					
12	0.881	0.882	0.824	0.732	0.734	1.011	0.935	0.794	0.781	1.163	1.004	0.000				
13	0.780	0.859	0.779	0.697	0.745	0.808	0.884	0.682	0.699	1.218	1.019	0.217	0.000			
14	0.564	0.952	0.676	0.489	0.567	0.779	0.854	0.584	0.603	0.878	0.903	0.618	0.433	0.000		
15	0.570	0.950	0.692	0.575	0.660	0.696	0.852	0.611	0.675	0.914	0.954	0.633	0.426	0.003	0.000	
16	0.875	1.097	0.922	0.788	0.877	0.874	1.076	0.855	0.903	1.129	1.134	0.731	0.512	0.093	0.029	0.000

* 1–11 are populations distributed in the Bayinbuluke area; 12 is a population distributed in the Tianchi area; 13 is a population distributed in the Turpan area; 14–16 are populations distributed in the Hami region.

**Table A3:** Topographic factor information.

Variable Code	Unit	Description
Latitude	°	Latitude
Longitude	°	Longitude
Podu	°	Gradient
Poxiang	-	Aspect
Soil_ph	-	Soil PH value (×10)
Clay	%	Percentage of clay in soil
Clcd	-	China Land Cover Dataset
Ndvi	-	Normalized Difference Vegetation Index
River	-	Euclidean distance from the river

**Table A4:** Bioclimatic factor information.

Variable Code	Unit	Description
Bio2	°C	Average daily temperature range (annual average)
Bio4	-	Temperature seasonality (standard deviation × 100)
Bio9	°C	The average temperature of the driest quarter
Bio13	mm	Wet month precipitation
Bio14	mm	Precipitation in the driest month
Bio15	-	Precipitation season (coefficient of variation)
Srad_01	kJ m^−2^ d^−1^	Solar radiation in January
Srad_07	kJ m^−2^ d^−1^	Solar radiation in July
Wind_01	M·s^−1^	January wind speed
Wind_07	M·s^−1^	July wind speed
Vapr_01	kPa	January water vapor pressure
